# Assessment of the ileoanal pouch for the colorectal surgeon

**DOI:** 10.1007/s00423-023-03151-5

**Published:** 2023-11-01

**Authors:** Valerio Celentano, Carlo Alberto Manzo

**Affiliations:** Inflammatory Bowel Disease and Ileoanal Pouch Surgery Centre, Chelsea and Westminster Hospital, NHS Foundation Trust, 369 Fulham Road, London, UK

**Keywords:** Ileoanal pouch, Pouchoscopy, J-pouch, Inflammatory bowel disease, IPAA

## Abstract

**Introduction:**

Many pouch complications following ileoanal pouch surgery have an inflammatory or mechanical nature, and specialist colorectal surgeons are required to assess the anatomy of the ileoanal pouch in multiple settings. In this study, we report our stepwise clinical and endoscopic assessment of the patient with an ileoanal pouch.

**Methods:**

The most common configuration of the ileoanal pouch is a J-pouch, and the stapled anastomosis is more frequently performed than a handsewn post-mucosectomy. A structured clinical and endoscopic assessment of the ileoanal pouch must provide information on 7 critical areas: anus and perineum, rectal cuff, pouch anal anastomosis, pouch body, blind end of the pouch, pouch inlet and pre-pouch ileum.

**Results:**

We have developed a structured pro forma for step-wise assessment of the ileoanal pouch, according to 7 essential areas to be evaluated, biopsied and reported. The structured assessment of the ileoanal pouch in 102 patients allowed reporting of abnormal findings in 63 (61.7%). Strictures were diagnosed in 27 patients (26.4%), 3 pouch inlet strictures, 21 pouch anal anastomosis strictures, and 3 pre-pouch ileum strictures. Chronic, recurrent pouchitis was diagnosed in 9 patients, whilst 1 patient had Crohn’s disease of the pouch.

**Conclusions:**

Detailed clinical history, assessment of symptoms and multidisciplinary input are all essential for the care of patients with an ileoanal pouch. We present a comprehensive reporting pro forma for initial clinical assessment of the patient with an ileoanal pouch, with the aim to guide further investigations and inform multidisciplinary decision-making.

## Introduction

Ileal pouch-anal anastomosis (IPAA) following restorative proctocolectomy is the surgical procedure of choice for ulcerative colitis (UC) and familial adenomatous polyposis (FAP), as an alternative to permanent end ileostomy and when ileorectal anastomosis is not appropriate. The multidisciplinary management for ileoanal pouch patients involves dedicated colorectal surgeons and gastroenterologists throughout the course of the disease, working synergistically with specialist nurses, radiologists and dietitians, to maintain a healthy pouch and to treat short- and long-term complications [1].

Many pouch complications have an inflammatory or mechanical nature and can be successfully treated if promptly diagnosed. The ability to perform an endoscopic assessment of the pouch “pouchoscopy” is an essential skill required of the pouch surgeon, who will be reviewing pouch patients in the outpatient clinic or in the operating theatre under general anesthetics. Specialist colorectal surgeons are required to assess the anatomy of the ileoanal pouch in multiple settings: right after the ileal pouch-anal anastomosis fashioning, during surveillance endoscopic follow-up, to detect inflammatory disorders of the pouch and to exclude any mechanical conditions associated with pouch dysfunction [2].

Gaining experience in pouch surgery is difficult as the procedure is performed infrequently across many hospitals, as evident from the UK Pouch registry, reporting that the average number of pouches performed in English institutions was just three cases per year, and one quarter of the pouch surgeons undertaking this surgery had performed only one case over the last 5 years [3].

In this study, we report our stepwise clinical and endoscopic assessment of the patient with an ileoanal pouch and present a dedicated reporting pro forma we have developed.

## Methods

**Fig. 1:**
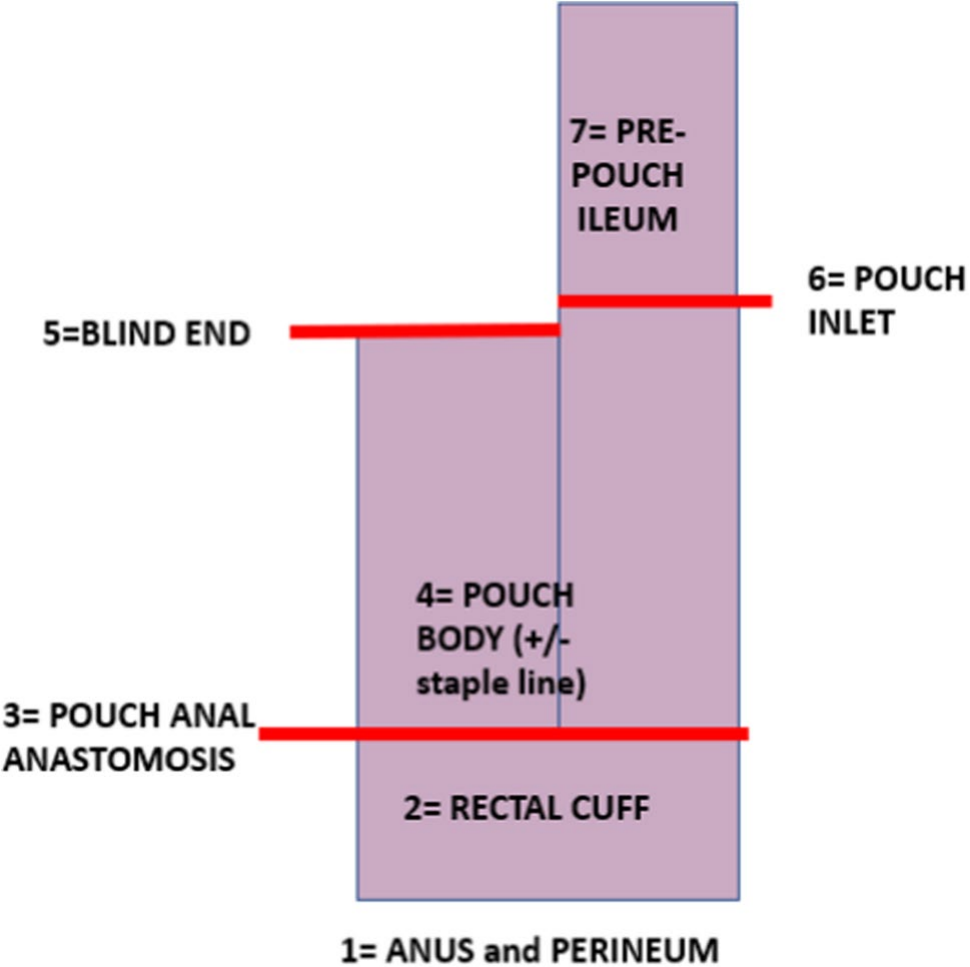
Anatomy of the ileoanal pouch

The most common configuration of the ileoanal pouch is a J-pouch (Figure 1), and the stapled anastomosis is more frequently performed than a handsewn post-mucosectomy. Pouch surgeons need to be familiar with other pouch configurations such as the S- and W-pouch. Prior to pouchoscopy, an enema will be administered in the endoscopy department, and in most of the patients, the pouchoscopy is performed under sedation, preferring the use of a paediatric colonoscope or of a gastroscope in patients with strictures.

In view of the complexity of the pouch anatomy, we believe that a structured clinical and endoscopic assessment of the ileoanal pouch must provide information on 7 critical areas.

### Step 1: anus and perineum

The observation of anal and perianal skin irritation can be an indirect sign of poor pouch function. The perianal region is inspected for previous surgical incisions and scars and for signs of fistulating disease or collections. The presence and location of external openings is noted, and inspection of the skin extending towards the scrotum and groins is essential, as it is to perform a vaginal examination to rule out pouch-vaginal fistulae.

Anal canal stricture is a common condition after an IPAA, usually diagnosed with digital examination and requiring dilation. According to Prudhomme, the anal strictures can be subdivided into non-rigid or rigid depending on the presence of palpable fibrosis [4].

The anal transition zone (AZT) is an area in the anal canal between the squamous epithelium of the anoderm and dentate line below and the uninterrupted rectal columnar epithelium above; it extends for a variable length (typically 1–2 cm) and is best represented in stapled anastomosis. Along with the retained rectal cuff, it has a potential risk for inflammation, dysplasia and cancer [5].

This step of pouch assessment is completed by evaluating the tone of the anal sphincter complex and the squeeze pressure at digital rectal examination. The presence of anal canal problems, such as fissures, skin tags and haemorrhoids, is also reported.

### Step 2: rectal cuff

The inflammation of the rectal cuff or cuffitis is one of the long-term complications of IPAA. Correct division of the rectum at the level of the levators remains a significant challenge in minimally invasive pouch surgery, with inappropriately retained long rectal cuffss, leading to bleeding, tenesmus, urgency and a risk of dysplasia or cancer.

Rectal examination allows for detection of any intra-anal-rectal lesions, appreciating the distance of the anastomosis from the anal verge. Particularly during intraoperative evaluation, care must be taken in evaluating possible discrepancies in the length of rectal cuff, which can be left longer anteriorly and shorter posteriorly, as for the possible presence of “dog-ears” remnants of the double stapled anastomosis.

### Step 3: ileal pouch anal anastomosis

Up to 15% of ileoanal pouches will develop a symptomatic leak [6]. The clinical presentation is driven by the timing of the leak. Acute or early postoperative leaks usually present with pelvic sepsis and systemic symptoms. Shen classified the most frequent leak sites as the pouch-anal anastomosis, the blind end and the vertical staple line of pouch body. IPAA leaks are frequent, and sometimes, small leaks can go unnoticed due to the presence of a diverting loop ileostomy; nevertheless, these can hesitate in chronic sinuses and/or fistulae and ultimately lead to pouch failure [7]. For this purpose, routine postoperative soluble contrast enema studies, or even better magnetic resonance imaging (MRI), can help reveal clinically silent leaks and avoid chronic complications.

A pouch sinus is another late presentation of an undetected IPAA anastomotic leak, and it is described as a blind-ended pouch tract connected to a pelvic abscess. This condition affects from 3 to 8% of patients undergoing IPAA and when not timely treated leads to pouch failure in one patient out of 3 [8]. The most common location for this condition is usually the posterior aspect of the IPAA, at the presacral space. Coccygeal pain can be reported in patients developing osteomyelitis.

Fistulas at the IPAA can involve the vagina, skin, urethra, prostate or gluteal muscles such as in pouch-vaginal or pouch-cutaneous fistula, the two most common types. Multiple classifications for pouch fistulas have been proposed, based on etiology, location of the primary orifice or target organ. The height of the internal opening can guide the differentiation between anastomotic dehiscence and cryptoglandular which are typically below the anastomosis.

The diagnosis of pouch fistula is mainly based on patient’s symptoms and clinical history, and suspect should always lead the surgeons to perform an evaluation under anesthesia (EUA) with pouchoscopy and a pelvic MRI to delineate the anatomy and evaluate possible undrained abscesses in the area. Pouch-vaginal fistula (PVF) is a challenging complication with devastating effect on women quality of life, not as rare since it has been reported in up to 16% of pouch patients and represents one of the main causes leading to pouch failure [9].

IPAA strictures are among the most common sequelae of pouch surgery, occurring in around 11–12% in UC and FAP patients. Strictures in pouch surgery may happen in multiple sites, like at the IPAA, at the mid-pouch or pouch body, at the pouch inlet and in the afferent limb of the pouch. The presence of a stricture at the IPAA level or lower may impair the possibility to perform pouchoscopy.

In the S-pouch configuration, an efferent limb will connect the pouch body to the ileaoanal pouch anastomosis, and its length needs to be reported, as limbs longer than 2 cm can be responsible for obstructed defecation or efferent limb syndrome.

### Step 4: pouch body

The pouch body is the main segment of the J-pouch in terms of volume, enclosed between the pouch anal anastomosis (pouch outlet) and the pouch inlet. Right after exploring the anastomosis, the endoscope will reach the pouch body, and by insufflating, it will be possible to assess the size and distensibility of the pouch. Pouch compliance is among the most important features for pouch function, being necessary for its activity as a reservoir for stool and its ability to empty during the defecation. A severely contracted pouch can be due to pelvic sepsis, or occasionally to a low volume pouch at the time of construction. Conversely, a large and dilated pouch can be the result of chronic obstruction, leading to incomplete defecation and need for self-catheter insertion.

**Fig. 2:**
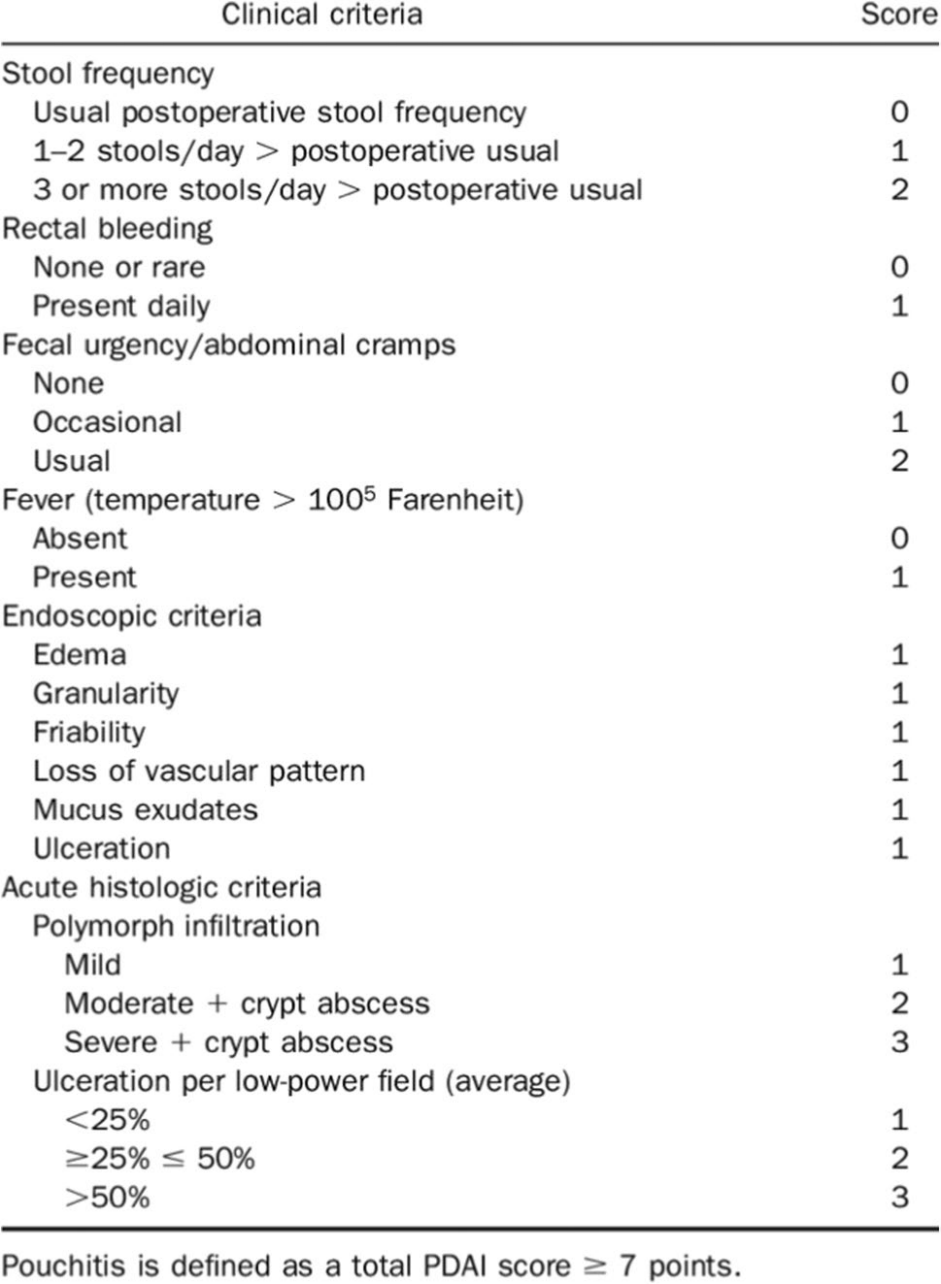
Pouchitis disease activity index

Pouch inflammation or Pouchitis is more common in UC than in FAP, with about 25 to 50% of pouch patients experiencing at least one episode in the first 10 years from the index surgery. Diagnosis and assessment rely on pouchoscopy and biopsies, with scores as the Pouchitis Disease Activity Index (PDAI, Figure 2) and its further versions (modified PDAI or mPDAI) [10] which rely on clinical, endoscopic and histological features.

It is a good practice to report the location of mucosal abnormalities in the body of the pouch, as ischaemic conditions tend to prefer the staple line or the distal third of the pouch body. When the vertical staple line of the pouch body is still visible, it can be used as landmark to assess obvious changes in the pouch orientation, which could be due to a twist in the pouch body or mesentery, leading to mechanical obstruction. Strictures of the pouch body are rare, as a sequela of severe and recurrent pouchitis or even of Crohn’s disease of the pouch.

### Step 5: blind end of the pouch

The blind end of a J-pouch corresponds to the stapled efferent limb, usually measuring around 1 to 2 cm. A dilated blind end can facilitate stool accumulation and prevent the pouch from completely emptying during evacuation or can bend and compress the afferent limb of the pouch resulting in a mechanical obstruction. Among the possible J-pouch leaks, the leak from the tip of the J is the second most common, with an incidence of 1% of pouch patients or approximately 15% of all the postoperative pouch leaks [11].

### Step 6: the pouch inlet

The pouch inlet represents the entrance door for the bowel content to the ileoanal pouch. During endoscopic assessment of the J pouch, the pouch inlet is visible as part of one of the two “owl’s eyes.” Ease of reaching such inlet can reveal much about the pouch structure, any anomalies in terms of shape or floppiness and any distortion due to adhesions. Ability and easiness of intubating the pouch inlet must be noted. Among the possible site of strictures in pouch surgery, which occur in up to almost 40% of all pouch patients, the pouch inlet is the second site, with the first being the pouch outlet. Inlet strictures can cause bowel obstruction, chronic evacuation problems with abdominal distension, pouch dilation with loss of function and pouchitis due to bacterial overgrowth. All pouch strictures must be biopsied, and their length noted. The ability to intubate with an adult colonoscope or gastroscope should be reported.

### Step 7: the pre-pouch ileum

The pre-pouch ileum is intubated to investigate mechanical (afferent limb acute angle, prolapse or intussusceptions) or inflammatory (pre-pouch ileitis, stricture or polyposis) pouch problems.

Pre-pouch ileitis (PI) was first described in 1994 as an inflammatory complication targeting the distal afferent limb of the J-pouch, with a reported incidence of around 5% [12]. Pouchoscopy will show features closely resembling CD, climbing the neo-terminal ileum with erosions, ulcerations, erythema and friability for up to 40–50 cm from the inlet. Any case of ileitis in the afferent limb must be investigated to rule out Crohn’s disease (CD), even though literature agreed PI should be considered a separate condition [13]. Recurrent or chronic PI may cause the growth of inflammatory polyps in the pre-pouch ileum that need to be biopsied or removed during surveillance pouchoscopy.

### Assessment of pro forma validity

Face and content validity and ease of use of the developed pro forma were evaluated by survey feedback from colorectal surgeons, gastroenterologists and trainees. Construct validity for the presence of strictures was obtained by comparing the structured assessment of the pouch with MRI imaging, when available. Completeness of the reporting by using the pro forma was evaluated by identifying the number of items documented on pouchoscopies reports retrospectively available for the same patients, prior to the introduction of the pro forma (convergent validity).

## Results

We have developed a structured pro forma for step-wise assessment of the ileoanal pouch, according to 7 essential areas to be evaluated, biopsied and reported (Table 1).

**Table 1:**
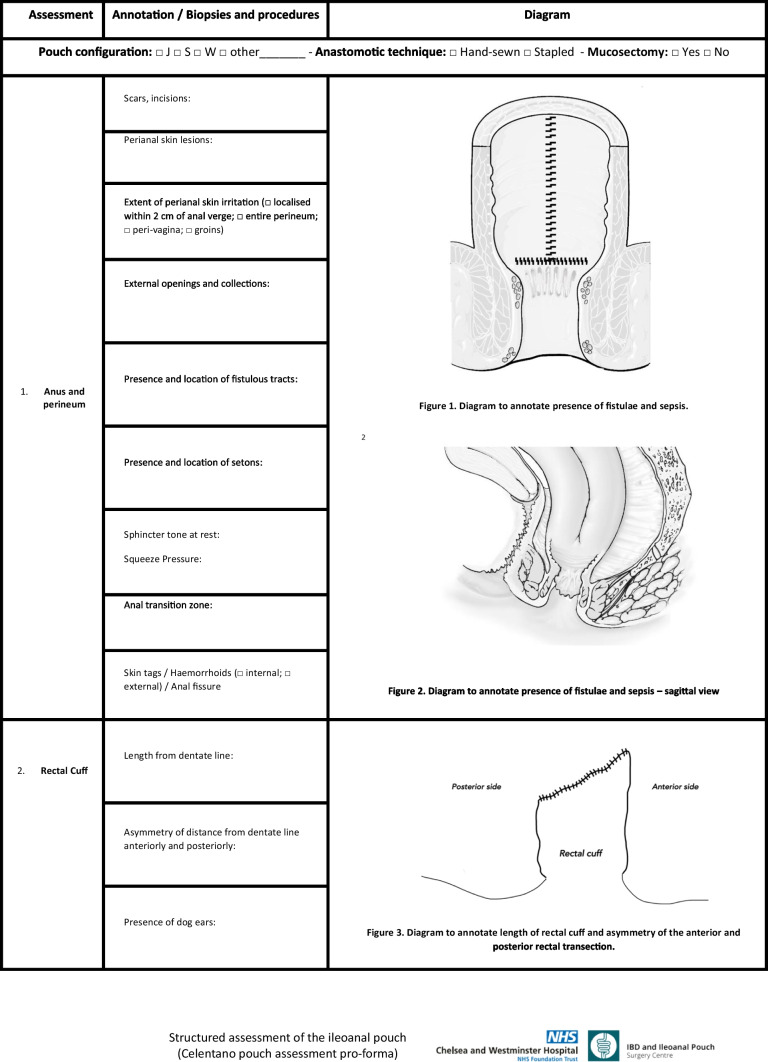

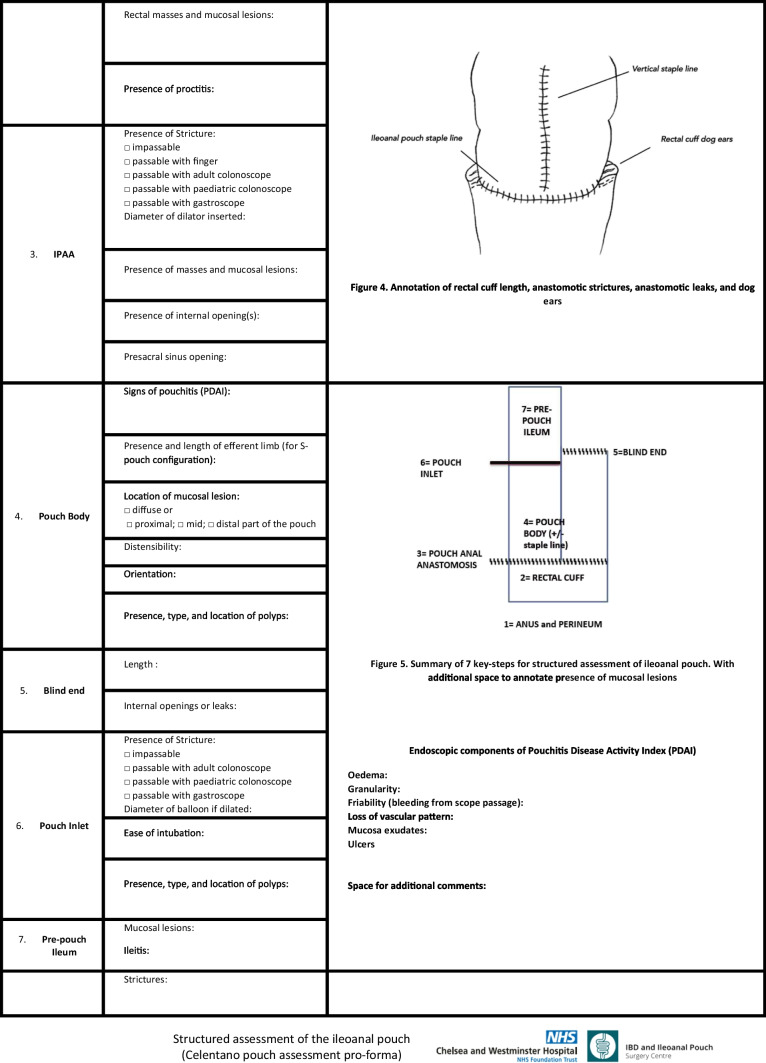
Structured assessment of the ileoanal pouch

We have applied the reporting pro forma in all the new referrals to our specialist ileoanal pouch clinic where we are able to offer multidisciplinary counselling, imaging and endoscopic procedures on the same appointment [14]. The “one-stop j-pouch clinic” is reserved for new referrals of patients considering having IPAA surgery or with pouch dysfunction. The referrals are triaged prior to booking, and further information from the referrer are requested, when needing to clarify the indication for same day investigations. Following the initial appointment, ongoing follow-up is organised in dedicated colorectal, gastroenterology and nurse-led clinics.

From August 2020 to March 2023, we have evaluated 102 patients with our structured clinical and endoscopic assessment, with all procedures performed by the same colorectal surgeon. Approximately 35% of the referrals originated from outside the Trust (tertiary referrals). The structured assessment of the ileoanal pouch allowed reporting of abnormal findings in 63 patients (61.7%). Strictures were diagnosed in 27 patients (26.4%), 3 pouch inlet strictures, 21 pouch anal anastomosis strictures and 3 pre-pouch ileum strictures. Afferent limb syndrome due to adhesions at the level of the pouch inlet was found in 1 patient. Chronic, recurrent pouchitis was diagnosed in 9 patients (8.8%), whilst 1 patient had Crohn’s disease of the pouch.

Fistulae were present in 7 patients (6.8%): 3 pouch-vaginal fistulae, 4 pouch anal fistulae. Pouch anal anastomotic leak was diagnosed in 4 patients (3.9%), whilst 2 patients had a chronic pouch sinus, and 1 patient had a chronic leak at the blind end of the pouch.

A rectal cuff longer than 2 cm was diagnosed in 11 patients (10.1%), whilst cuffitis in 15 patients (14.7%). No cases of invasive cancer were diagnosed, whilst one patient had a rectal polyp with low grade dysplasia, and another had diffuse high-grade dysplasia in the rectal cuff and anal transition zone.

The pro forma demonstrated acceptable face and content validity. Two gastroenterologists, 2 colorectal surgeons and 3 surgical trainees were surveyed on the use of the newly developed pouch pro forma for reporting of the clinical and endoscopic ileoanal pouch assessment. The median time needed to complete the report was 5 minutes (range 3–10), with all users satisfied that all required items were present in the pro forma. Validity of the test was confirmed by the fact that all strictures in the pre-pouch ileum and pouch inlet were also detected by MRI imaging, whilst of the 21 anastomotic strictures, only 17 (81%) were identified at MRI. Of the 102 patients included in the study, only 19 had a retrospective pouchoscopy report available.

## Discussion

We present a comprehensive reporting pro forma for initial clinical assessment of the patient with an ileoanal pouch, with the aim to guide further investigations and inform timely multidisciplinary decision-making. Detailed clinical history, assessment of symptoms and multidisciplinary input are all essential for the care of patients with an ileoanal pouch. Similar to inflammatory bowel diseases, diagnosis of pouch disorders is multifactorial and must involve clinical, radiological, endoscopic and pathological results. Clinical assessment and pouchoscopy should not be used in isolation but complementarily to all these other diagnostic tools. Nevertheless, pouchoscopy has a crucial role in intraoperative decision-making and in detecting complications of the ileoanal pouch that can benefit from surgical treatment. Our study provides a pro forma to help colorectal surgeons investigating pouch disorders, with the aim to minimise unstructured reporting. Unfortunately, it does not provide a score able to stratify the severity of the pouch complications or the response to treatment. We believe that a structured assessment of the ileoanal pouch must report routinely on both positive and negative findings, allowing retrospective review of patients who develop new symptoms during the long-term follow-up. Such a structured report could also facilitate inter-hospital communication, as not infrequently pouch patients can be followed in a tertiary centre away from the local hospital.

We routinely inspect intraoperatively the ileoanal pouch during restorative surgery, obtaining information on the completeness of the anastomosis, the presence of bleeding (allowing endoscopic haemostasis) or the suspect of ischaemic changes with the aid of indocyanine green. It is also a helpful feedback for direct visualisation of the dentate line to orientate if the appropriate level of rectal transection has been achieved. We also perform an intraoperative pouchoscopy at the time of the loop ileostomy reversal, despite preoperative contrast studies, due to the known risk of missing subclinical leaks or strictures.

There are known technical challenges surrounding the IPAA procedure, with significant focus around minimally invasive approaches, plane of rectal dissection and 3-stage versus 2-stage (and modified 2-stage) restorative proctocolectomy. These challenges are reflected in inconsistencies in IPAA surgery practice, with need for consensus on what is considered safe and appropriate [15].

Our study represents a single pouch centre experience, and the validity of our results, and replicability of the pouch reporting pro forma, must be further evaluated. Comparing the completeness of pouchoscopy reports since the introduction of the reporting tool would have also been deemed necessary but was not possible in our cohort due to the limited number of reports available retrospectively. However, we correlated the results of our clinical and endoscopic assessment with the imaging findings in the patients who had an MRI of the pouch available. Whilst our pouch assessment reporting pro forma is not yet validated for pouch assessment by multiple specialists or different hospitals, we believe that providing a routine structured framework for description of findings in pouch patients could lead to standardisation of reports, with more complete information available to clinicians.

## Data Availability

The datasets generated during and/or analysed during the current study are available from the corresponding author on reasonable request.
